# The impact of work interference with family on depressive symptoms among married working women: A longitudinal panel study

**DOI:** 10.1371/journal.pone.0276230

**Published:** 2022-11-09

**Authors:** Il Yun, Yun Hwa Jung, Eun-Cheol Park, Sung-In Jang

**Affiliations:** 1 Department of Public Health, Graduate School, Yonsei University, Seoul, Republic of Korea; 2 Institute of Health Services Research, Yonsei University, Seoul, Republic of Korea; 3 Department of Preventive Medicine, Yonsei University College of Medicine, Seoul, Republic of Korea; Fukuoka University, JAPAN

## Abstract

**Purpose:**

This study aimed to investigate the effects of work interference with family (WIF) on depressive symptoms among married working women.

**Methods:**

Data from 2014‒2018 of the Korean Longitudinal Survey of Women and Families were used. Only married women living with their husbands and wage workers were included, whereas those diagnosed with depression at the baseline year and those with missing values were excluded. A total of 1,504 individuals were included as the study population. The impact of WIF on depressive symptoms was analyzed using the generalized estimating equation model with a logit link.

**Results:**

After adjusting for all the potential confounding variables, it was found that WIF had an effect on depressive symptoms. Women whose work interfered with their family were 1.58 times more likely to experience depressive symptoms than those who did not experience WIF (95% Confidence interval (CI): 1.30‒1.92). WIF due to irregular working hours had the highest odds ratio for depression (Adjusted odds ratio (OR): 2.01, 95% CI: 1.32‒3.08). Women with WIF were more likely to develop depressive symptoms when they had two or more children (With 2 children, Adjusted OR: 1.69, 95% CI: 1.31‒2.18; With 3 or more children, Adjusted OR: 1.63, 95% CI: 1.07–2.49).

**Conclusions:**

Thus, married working women who experienced WIF were found to be at a higher risk of developing depressive symptoms. Therefore, considering how prevailing stereotypes that roles of men and women are separated can harm women’s mental health, policy measures should be implemented to ensure women’s quality of life based on the work-family balance.

## Introduction

The Organisation for Economic Co-operation and Development (OECD) statistics reveal that the employment rate of women in Korea is about 57%. Korea ranks 36^th^ in this aspect among the 38 OECD countries [1]. In particular, it was found that career breaks occurred among women aged 35‒39 years [1]. This is an indication that women are excluded from the labor market for reasons such as childbirth and childrearing. In addition, the total fertility rate is the lowest among the OECD countries in Korea, with an average of 0.81 births per woman [2]. These issues have drawn the attention of the government’s social agenda to urgently consider women’s welfare policies at the national level, and resolve the extremely low fertility rates and women’s career breaks.

The issue of stereotyped gender roles at work and at home is not unique to Korea but common to most traditional East Asian family patterns and customs, where men work outside the home and women are engaged in child rearing and household chores [3]. Finding the balance between work and family and resolving work-family conflicts have long been topics of discussion in Western countries, which are free from the prejudice that men and women have different roles at work at home, and women’s labor-force participation is relatively high [4, 5]. However, in East Asia, these are recent trends. As women’s educational background and social status gradually increased in East Asia, there was a corresponding increase in the demand for reducing gender discrimination. Hence, conflicts over gender roles at work and at home have decreased significantly compared with the past [6].

Research shows that work-family conflict adversely affects women’s mental health [7, 8] and well-being [9]. However, most of these studies were conducted in the West, and few have attempted to examine the effect of work interference with family (WIF) on depressive symptoms by clarifying the cause of WIF. Further, no domestic study involving a stratification analysis based on the number of children considering Korea’s ultra-low fertility rate was conducted.

Therefore, using longitudinal panel data, this study aimed to identify the impact of WIF on depressive symptoms among married working women. Furthermore, based on the analysis results, the causes for career breaks and low fertility rates were investigated, and appropriate policy measures were suggested to improve work-family balance in Korean married working women.

## Materials and methods

### Data and study population

Data analyzed in this study were drawn from the Korean Longitudinal Survey of Women and Families (KLoWF). The KLoWF is a longitudinal panel survey using a stratified multistage sampling design based on the Korean Population Housing Census data. The Korean Women’s Development Institute conducts this survey every two years since the first and second survey in 2007 and 2008, respectively. To provide a basis for establishing policies to improve work-family balance, the KLoWF consists of questionnaire items seeking information about women’s overall life including family, work, and daily activities [10]. Ethical approval was not required to conduct this study, as KLoWF is anonymized data publicly available for scientific use [11].

**Fig 1:**
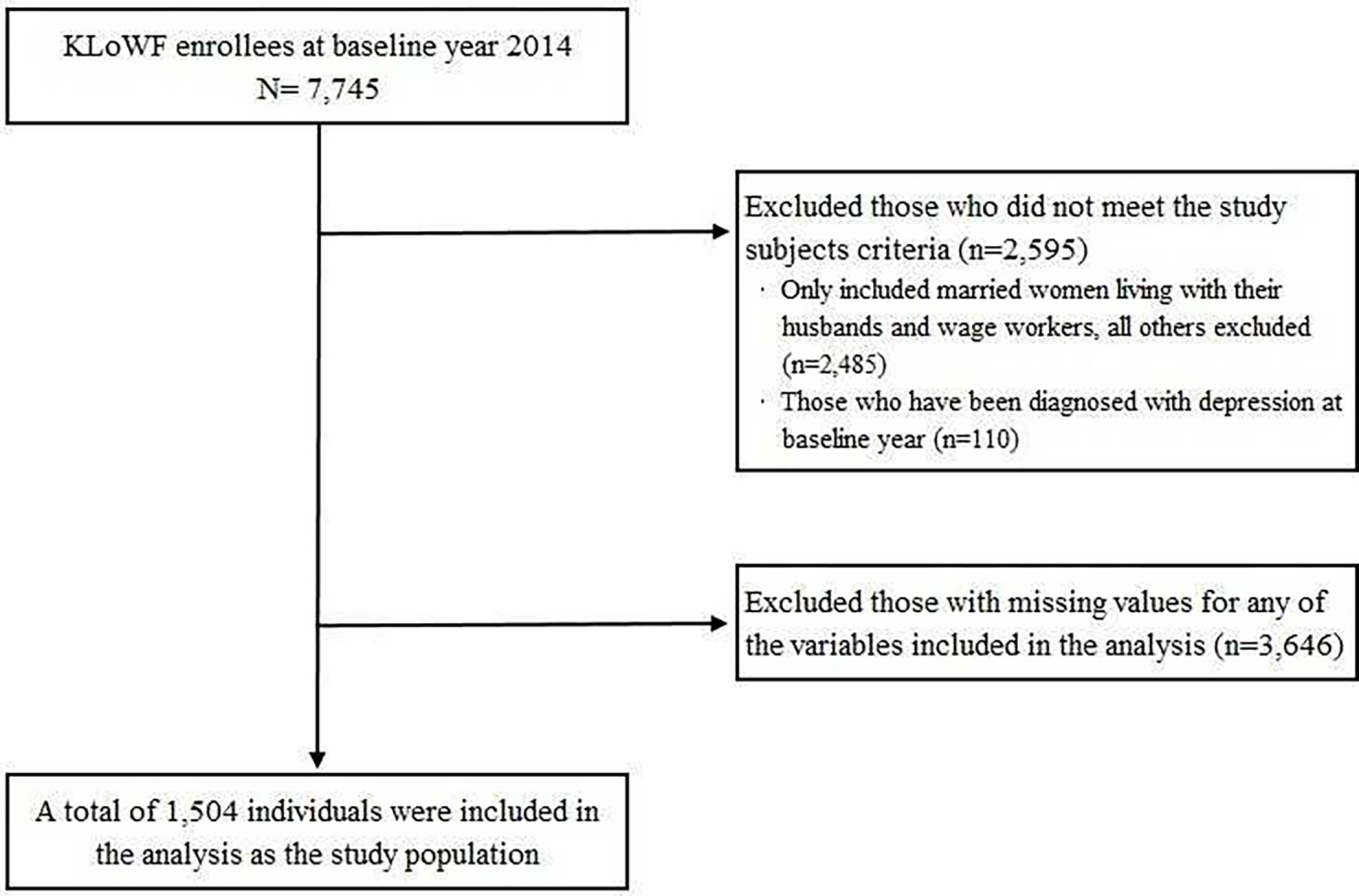
Flow chart of sample selection.

In this study, data from the fifth to seventh wave of KLoWF (2014‒2018) were used. In 2014, which was set as the baseline year, the number of survey participants was 7,745. Among them, for this study, only married women living with their husbands and wage workers were included, whereas others were excluded (n = 2,485). Then, based on the study hypothesis, those who were diagnosed with depression at the baseline year were excluded (n = 110). Finally, after excluding those with missing values (n = 3,646), 1,504 individuals were selected as the study population. The detailed flow of the sample selection is as depicted in Fig 1.

### Measures

The dependent variable was depressive symptoms, assessed using the 10-item version of the Center for Epidemiologic Studies Depression Scale (CES-D-10). The CES-D-10 is a simplified screening tool of CES-D, a 10-item Likert scale questionnaire assessing depressive symptoms if noticed in the week prior to the survey [12]. Of the 10 items, three items are about depressed affect, five about somatic symptoms, and two items about positive affect. For each item, respondents can select from “rarely or never” (score of 0) to “all the time” (score of 3), and only two items on positive affect statements were required to be reverse-coded. Finally, on a score of 0‒30, the higher the participants’ score, the more severe the depressive symptoms [13]. In this study, since depressive symptoms were analyzed as a categorical variable, we applied the cutoff score for indicating significant depressive symptoms if the score was greater than or equal to 10 out of 30 [12, 14].

As the main variable of interest, we used WIF to explain the work-family conflict of married working women [15]. Each participant was asked the following two questionnaire items about WIF to score on a 4-point scale: 1) “Working hours are too long, which interferes with family life,” 2) “Working hours are irregular, which interferes with family life.” Analyses were performed by categorizing 1 and 2 points as “agree,” and 3 and 4 points as “disagree.” Only the negative effects of work on the family were considered to examine the influence of WIF on depressive symptoms in married working women.

We controlled for potential confounding variable such as socioeconomic, health-related, and work-related factors. Socioeconomic factors included age (20~39, 40~49, 50~59, and 60 or older), region (urban and rural), highest level of education (middle school or below, high school, and college or above), and household income level. Health-related factors included perceived health status (good and bad), and number of children (0, 1, 2, and 3, or more). Factors related to work included, first of all, occupation. Occupations were classified into white-collar performing work in an office or administrative setting, pink-collar working in the care-oriented career field or in a field that was historically considered a woman’s job, and blue-collar performing manual labor. In addition to this, monthly salary level, which was divided into quartiles, considering quartile 4 to be the highest earner, working hours (≤ 40 hours and > 40 hours per week), and job satisfaction (satisfied and dissatisfied) were included as covariates.

### Statistical analysis

The chi-squared test was performed to investigate and compare the general characteristics of the study population, and the descriptive statistics were shown as frequencies (*N*) and percentages (%). Thereafter, the impact of WIF on depressive symptoms was analyzed using the generalized estimating equation (GEE) model with a logit link, which considers time variation and correlations between repeated measurements in a longitudinal study design [16]. The key results were presented as odds ratio (ORs) and 95% confidence intervals (CIs). For all analyses, we used SAS version 9.4 (SAS Institute Inc; Cary, NC, USA), and the statistical significance level was defined as *p*-value < .05.

## Results

Table 1 presents the general characteristics of the study population at the baseline year 2014. Of the 1,504 participants, 531 (35.3%) were identified to have experienced WIF. There was a statistically significant difference in the depressive symptoms according to the experience of WIF among married working women.

**Table 1:** General characteristics of the study population at baseline year (2014).

Characteristics				Depressive symptoms				
		Total		CES-D-10^d^ score ≥10		CES-D-10^d^ score < 10		*P-value*
		N	%	N	%	N	%	
		1,504	100.0	154	10.2	1,350	89.8	
**Work interference with family (WIF)**								0.003
	Yes	531	35.3	71	13.4	460	86.6	
	No	973	64.7	83	8.5	890	91.5	
**Age**								0.002
	20~39	334	22.2	33	9.9	301	90.1	
	40~49	710	47.2	57	8.0	653	92.0	
	50~59	338	22.5	40	11.8	298	88.2	
	60 or older	122	8.1	24	19.7	98	80.3	
**Region**								0.724
	Urban	928	61.7	93	10.0	835	90.0	
	Rural	576	38.3	61	10.6	515	89.4	
**Highest level of education**			0.0					0.002
	Middle school or below	223	14.8	37	16.6	186	83.4	
	High school	667	44.3	65	9.7	602	90.3	
	College or above	614	40.8	52	8.5	562	91.5	
**Number of children**								0.374
	0	81	5.4	7	8.6	74	91.4	
	1	180	12.0	24	13.3	156	86.7	
	2	923	61.4	87	9.4	836	90.6	
	3 or more	320	21.3	36	11.3	284	88.8	
**Household income level**								< .0001
	Quartile 1 (low)	391	26.0	66	16.9	325	83.1	
	Quartile 2	362	24.1	39	10.8	323	89.2	
	Quartile 3	376	25.0	25	6.6	351	93.4	
	Quartile 4 (high)	375	24.9	24	6.4	351	93.6	
**Occupation**								0.050
	White-collar	685	45.5	56	8.2	629	91.8	
	Pink-collar	425	28.3	52	12.2	373	87.8	
	Blue-collar	394	26.2	46	11.7	348	88.3	
**Monthly salary level**								0.007
	Quartile 1 (low)	335	22.3	50	14.9	285	85.1	
	Quartile 2	441	29.3	46	10.4	395	89.6	
	Quartile 3	376	25.0	30	8.0	346	92.0	
	Quartile 4 (high)	352	23.4	28	8.0	324	92.0	
**Working hours**								0.805
	≤ 40 hours per week	1,087	72.3	110	10.1	977	89.9	
	> 40 hours per week	417	27.7	44	10.6	373	89.4	
**Job satisfaction**								0.001
	Satisfied	931	61.9	118	12.7	813	87.3	
	Dissatisfied	573	38.1	36	6.3	537	93.7	
**Perceived health status**								< .0001
	Good	1,008	67.0	74	7.3	934	92.7	
	Bad	496	33.0	80	16.1	416	83.9	

^d^CES-D-10: 10-item version of the Center for Epidemiologic Studies Depression Scale.

Table 2 shows the results of GEE analysis of factors associated with depressive symptoms. After adjusting for all the potential confounding variables, we found that WIF had an effect on depressive symptoms. Those whose work interfered with their family were 1.58 times more likely to suffer from depressive symptoms than those who did not experience WIF (95% CI: 1.30‒1.92). In addition, job satisfaction and perceived health status were found to be factors associated with depressive symptoms.

**Table 2:** Results of the GEE^a^ analysis of factors associated with depressive symptoms.

Variables		Depressive symptoms	
		Adjusted OR^b^	95% CI^c^
**Work interference with family (WIF)**			
	Yes	1.58	(1.30–1.92)
	No	1.00	
**Age**			
	20~39	1.00	
	40~49	0.99	(0.73–1.33)
	50~59	1.37	(0.98–1.92)
	60 or older	1.29	(0.81–2.06)
**Region**			
	Urban	1.00	
	Rural	0.80	(0.64–0.99)
**Highest level of education**			
	Middle school or below	1.11	(0.74–1.65)
	High school	1.00	(0.76–1.31)
	College and above	1.00	
**Number of children**			
	0	1.00	
	1	1.02	(0.61–1.72)
	2	0.94	(0.59–1.51)
	3 or more	1.06	(0.65–1.75)
**Household income level**			
	Quartile 1 (low)	1.86	(1.35–2.55)
	Quartile 2	1.26	(0.91–1.75)
	Quartile 3	1.09	(0.79–1.50)
	Quartile 4 (high)	1.00	
**Occupation**			
	White-collar	1.00	
	Pink-collar	0.96	(0.72–1.27)
	Blue-collar	0.69	(0.50–0.95)
**Monthly salary level**			
	Quartile 1 (low)	1.42	(0.97–2.07)
	Quartile 2	1.36	(0.95–1.96)
	Quartile 3	1.29	(0.90–1.85)
	Quartile 4 (high)	1.00	
**Working hours**			
	≤ 40 hours per week	1.00	
	> 40 hours per week	0.96	(0.75–1.24)
**Job satisfaction**			
	Satisfied	1.00	
	Dissatisfied	1.56	(1.25–1.94)
**Perceived health status**			
	Good	1.00	
	Bad	1.65	(1.36–2.01)

^a^GEE: Generalized estimating equation

^b^OR: odds ratio

^c^CI: confidence interval.

**Fig 2:**
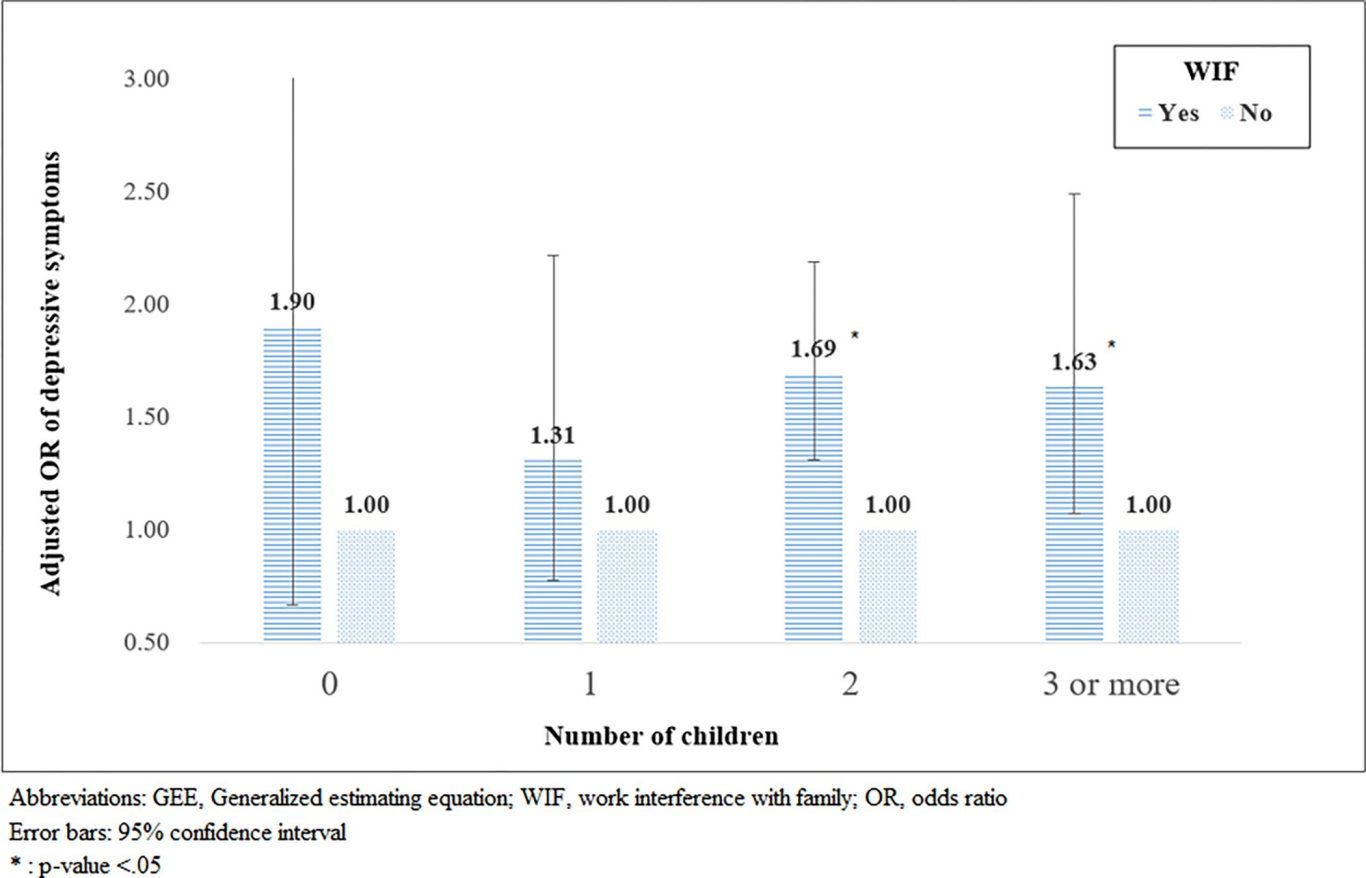
Results of subgroup analysis using GEE stratified by the number of children. Abbreviations: GEE, Generalized estimating equation; WIF, work interference with family; OR, odds ratio. Error bars: 95% confidence interval. *: p-value < .05.

Since the effect of WIF on depressive symptoms was assumed to vary depending on the number of children, a subgroup analysis was performed using GEE stratified by the number of children. The results in Fig 2 show that the ORs of the group that responded that they experienced WIF were higher than one regardless of the number of children; however, statistical significance was found only in the group with two children and the group with three or more children. Women with two children, who experienced WIF, were 1.69 times more likely to feel depressed than those who did not experience WIF (95% CI: 1.31–2.18). Women with three or more children, who complained of WIF, were 1.63 times more likely to have depressive symptoms than those who did not experience WIF (95% CI: 1.07–2.49).

Additionally, stratified subgroup analyses were conducted for each of WIF and for severity of depression; the results of these are presented in Tables 3 and 4, respectively. Those who mentioned both long and irregular working hours as reasons for WIF were 1.71 times more likely to experience depressive symptoms than those who did not experience WIF at all (95% CI: 1.35–2.16). In contrast, women who complained of WIF only because of irregular working hours show a higher OR to feel more depressed (Adjusted OR: 2.01, 95% CI: 1.32–3.08). Lastly, in the subgroup analysis according to the severity of depression, considering those who did experience WIF and did not have depressive symptoms as a reference, when WIF was experienced, the OR for mild depressive symptoms was 1.55 (95% CI: 1.25–1.93), and the OR for severe depressive symptoms was 1.79 (95% CI: 1.21–2.66).

**Table 3:** Results of subgroup analysis stratified by WIF.

Questionnaire items regarding WIF^a^		Depressive symptoms	
		OR^b^	95% CI^c^
Overly long working hours, interfering with family life	Irregular working hours interfering with family life		
Yes	No	1.40	(1.06–1.85)
No	Yes	2.01	(1.32–3.08)
Yes	Yes	1.71	(1.35–2.16)
No	No	1.00	

^a^WIF: work interference with family; ^b^OR: odds ratio; ^c^CI: Confidence Interval.

ORs were adjusted for other covariates, respectively.

**Table 4:** Results of subgroup analysis stratified by severity of depression.

WIF^a^	Depressive symptoms											
	Normal		Mild					Severe				
	(CES-D-10^d^ score < 10)		(10 < CES-D-10^d^ score ≤ 14)					(CES-D-10^d^ score > 14)				
	N	%	N		%			N		%		
	1350	89.8	123		8.2			31		2.1		
	OR^b^		OR^b^	95% CI^c^				OR^b^	95% CI^c^			
Yes			1.55	(1.25–1.93)				1.79	(1.21–2.66)			
No	1.00											

^a^WIF, work interference with family

^b^OR, odds ratio

^c^CI: Confidence Interval

^d^CES-D-10: 10-item version of the Center for Epidemiologic Studies Depression Scale.

ORs were adjusted for other covariates, respectively.

## Discussion and conclusions

Although there is no distinction between gender roles at work and at home, as compared to the past, it is still not easy for working women to achieve the work-family balance owing to life experiences such as pregnancy, childbirth, and child rearing. In light of this, this longitudinal panel study aimed to explore the impact of WIF on depressive symptoms in Korean married working women. The findings were not surprising: women who believed that their work interfered with their families were more likely to have depressive symptoms.

In addition, it was found that working women with two or more children were clearly more likely to have depressive symptoms if they experience WIF. Although the number of children did not have a direct effect on depression, our findings suggest that the number of children and WIF experience combined could significantly affect the mental health of working married women. Therefore, policies for women in the future should not be merely a means to increase the fertility rate, but also to ensure that women can continue to work despite having children, and that work-family conflict does not harm their mental health. There is a need to provide counseling support to women who experience depression due to WIF. Furthermore, a noteworthy finding is that the OR value for depression was particularly high when irregular working hours were included in the reason for WIF. In this regard, additional analysis was conducted, and the results showed that women with many children or low monthly salaries mainly complained of irregular working hours as the reason for WIF. This indicates that a woman with a child is being forced to work as a non-regular or part-time worker with low wages, thus leading to WIF. Therefore, interventions are needed to allow married women with children to have more flexible working hours.

Numerous studies in the West have examined the relationship between married working women’s work-family balance and their well-being [3]. They have pointed out that work-family conflict is an important stressor that affects professional women’s mental health [9, 17, 18]. In addition, studies have also revealed that work-family conflict affects both mental and physical health [19]; some have investigated the effect of gender differences in perceptions about work-family conflict on depression [20]. However, since traditional East Asian families follow the typical culture of husbands working outside and wives raising the children or performing household chores, studies on married working women are limited [21]. Over time, preconceived notions that gender roles are segregated have been broken in East Asia, and similar studies on the quality of life and health of married working women are now being conducted. For example, work-family conflict and husband’s support have been reported as factors influencing the stress of married working women [22]. Similar to our study, studies have also investigated the association between work-family conflict and depression in professional women [3, 23]. Consistent with findings from previous studies examining the effects of work-family conflict on depression and mental health, our study also found that married working women who experienced WIF. In addition, we performed a subgroup analysis of the poor working conditions that caused the WIF, and confirmed that, in particular, those who complained of WIF due to irregular working hours tended to become more depressed.

A major strength of our findings is that they were derived through analysis of panel data representing domestic women, and hence, could be generalized to the national level. Panel data also has the advantage of being applicable to policy reform by continuous follow-ups in the future. However, an important limitation of this study is that we defined work-family conflict by limiting it to WIF to examine only the negative effects of work life on family life. Although there is family interference in work (FIW) [24, 25], the inverse of WIF, we only considered WIF as the main interest of variable to lay the groundwork for devising policies for better working conditions for married women. For a more balanced work-family life, future studies must identify factors affecting FIW. Second, we investigated the impact of WIF only on depressive symptoms, a representative indicator of mental health, and did not explore other effects on physical health. This was because the KLoWF data we analyzed mainly consists of items on women’s daily life, work, and well-being; hence, it was not possible to examine the effect of WIF on physical health. Third, we used only two questionnaires to score the WIF, but there will be other poor working conditions that interfere with family life, in addition to long working hours and irregular working hours. Due to the limitations of the data used, WIF caused by other reasons could not be considered. Lastly, we tried to adjust for potential confounders that may affect WIF and depressive symptoms; however, we cannot rule out residual confounding affects from unmeasured variables.

In conclusion, our findings demonstrated that married working women who experienced WIF are more likely to feel depressed. In particular, a higher risk was found when a female worker with two or more children experienced WIF, and she complained of WIF due to irregular working hours. These results suggest that married working women are not yet completely free of stereotyped gender roles, thus leading to work-family conflicts and ultimately affecting their mental health. Appropriate policy measures should be taken to alleviate WIF and ensure women’s quality of life based on work-family balance.
